# Research trends in post-ERCP pancreatitis: a dual-database bibliometric analysis

**DOI:** 10.3389/fmed.2026.1966046

**Published:** 2026-10-02

**Authors:** Wei Tian, Xu Deng, Chunyuan Yang, Jianxing Tian, Zonglong Zhu, Rui Huang, Wei Pan

**Affiliations:** Department of Hepatobiliary and Pancreatic Surgery, The People’s Hospital of Lezhi, Ziyang, China

**Keywords:** adverse events, artificial intelligence, endoscopic retrograde cholangiopancreatography, pancreatic stent, post-ERCP pancreatitis, prophylaxis, risk prediction

## Abstract

**Background:**

Endoscopic retrograde cholangiopancreatography (ERCP) is associated with several adverse events, of which post-ERCP pancreatitis (PEP) is the most frequent despite established preventive measures. Clinical reviews evaluate risk factors and interventions, but the organization and development of the research field have received less attention. This study examined the development of the literature on PEP incidence, risk stratification, prediction, and prevention.

**Methods:**

The Web of Science Core Collection (WoSCC) and Scopus were last searched on July 31, 2026. English-language articles and reviews were merged, deduplicated, screened, and manually standardized. The merged dataset was used to analyze publication output, countries, institutions, authors, source journals, and author keywords; citation-dependent analyses were restricted to WoSCC records. VOSviewer and CiteSpace were used for collaboration, co-occurrence, co-citation, clustering, temporal, and citation-burst analyses.

**Results:**

The final dataset comprised 1,686 publications, including 1,414 articles and 272 reviews; 1,449 WoSCC records were available for citation-dependent analyses. Annual publication was sporadic before 1996 and peaked at 133 records in 2025. The United States contributed 485 publications, followed by China (306) and Japan (217), and maintained links with countries across all three collaboration clusters. *Gastrointestinal Endoscopy* published the most records (*n* = 170) and was the most frequently co-cited journal (*n* = 11,065). Temporal analyses showed increasing attention to multimodal prevention and risk assessment. At the retrieval cutoff, adverse events and risk factors had the strongest ongoing keyword bursts, while artificial intelligence and machine learning remained emerging topics.

**Conclusion:**

PEP research has moved toward multimodal prevention and individualized risk estimation. The literature remains concentrated in specialist endoscopy journals and established clinical networks, with substantial contributions from East Asia. Broader multicenter collaboration and independent validation are needed before emerging prediction models can inform routine practice.

## 1 Introduction

Endoscopic retrograde cholangiopancreatography (ERCP) is widely used to treat biliary and pancreatic disorders but remains associated with procedure-related adverse events (1). Post-ERCP pancreatitis (PEP) is the most frequent of these complications. A meta-analysis of 145 randomized trials estimated PEP incidences of 10.2% in unselected patients and 14.1% in high-risk patients (2). Although most cases are mild, severe PEP can prolong hospitalization, require intensive care, and occasionally result in death. Its continuing clinical burden has sustained efforts to improve risk assessment and prevention.

The risk of PEP is shaped by patient susceptibility, the indication for ERCP, and events occurring during the procedure (3). Research in this field has consequently extended beyond the identification of individual risk factors. Current work encompasses patient selection, cannulation strategy, pharmacological prophylaxis, pancreatic stenting, intravenous hydration, and risk-guided prevention (4). The breadth of these topics has produced a large and clinically heterogeneous body of literature involving different interventions, study designs, professional groups, and research settings.

Systematic reviews and clinical guidelines synthesize intervention effects and inform clinical decisions, but they provide limited insight into how the research field itself has developed. Questions concerning the geographical distribution of research, patterns of institutional and author collaboration, the intellectual foundations of the field, and shifts in thematic emphasis require analysis at the literature level. Previous bibliometric work focused on the 100 most-cited ERCP publications (5), while a more recent study examined pancreatitis together with perforation and infection within the broader literature on post-ERCP complications (6). Neither study characterized the broader literature on PEP incidence, risk factors, prediction, and prevention across its historical development.

This study used a dual-database bibliometric and knowledge-mapping approach to examine literature on PEP incidence, risk stratification, prediction, and prevention. We characterized publication and collaboration patterns, identified the intellectual and thematic structure of the literature, and traced changes in research emphasis over time.

## 2 Materials and methods

### 2.1 Study design and reporting framework

This bibliometric and knowledge-mapping study examined literature on PEP, focusing on its incidence, risk factors, risk assessment, prediction, and prevention. Publication identification and selection were documented with reference to the Preferred Reporting Items for Systematic Reviews and Meta-Analyses 2020 statement (PRISMA 2020), and bibliometric methods were reported with reference to the BIBLIO guideline for biomedical bibliometric reviews (7, 8). The study protocol was not registered.

A formal risk-of-bias assessment was not performed because the study analyzed publication characteristics and knowledge structures rather than intervention effects. Potential bias arising from database coverage, duplicate records, indexing practices, and metadata ambiguity was mitigated through dual-database retrieval, cross-database deduplication, manual screening, and metadata standardization. Ethical approval and informed consent were not required because no human participants or identifiable patient information were involved.

### 2.2 Data sources and search strategy

The final searches of the Web of Science Core Collection (WoSCC; Clarivate) and Scopus (Elsevier) were conducted, and all records were exported, on July 31, 2026. No lower publication-year limit was applied.

Searches were conducted in the Topic field in WoSCC and the Title, Abstract, and Keywords fields in Scopus. Each search combined PEP-related terms with a second concept block covering incidence, risk assessment, prediction, and preventive interventions; records were required to contain terms from both blocks. This strategy was designed to identify clinically oriented research on PEP incidence, risk assessment, prediction, and prevention, rather than all aspects of PEP. Publications focused exclusively on cellular or pathophysiological mechanisms or management after PEP onset may not have been retrieved if they lacked terms from the second block in the searched fields. Records retrieved because of incidental mentions of these terms were subsequently assessed against the eligibility criteria described in Section 2.3. The complete database-specific strategies are provided in Supplementary Table S1.

Results were restricted to English-language articles and reviews. Editorials, letters, conference publications, corrections, notes, book chapters, and other document types were excluded. WoSCC records were exported as plain-text files with “Full Record and Cited References,” and Scopus records were exported in CSV format with all available bibliographic, affiliation, keyword, and cited-reference fields.

### 2.3 Data integration and study selection

Scopus records were converted into a WoS-compatible format and merged with the WoSCC records using CiteSpace version 7.0.1, following the general principles of cross-database metadata integration and deduplication described previously (9). Duplicates were identified from DOI, normalized title, publication year, and author information. Records with incomplete identifiers, minor title differences, or conflicting metadata were manually compared. When a publication was indexed in both databases, the WoSCC record was retained because its cited-reference fields were used for citation-dependent analyses. The merged dataset was then inspected for residual duplicates.

Publications were eligible when PEP was a substantive focus or a separately reported and interpretable outcome relevant to its incidence, risk factors, prediction, or prevention. Eligible domains included epidemiology, patient- or procedure-related risk factors, risk assessment or prediction, pharmacological prophylaxis, procedural or device-based prevention, and clinical guidance with substantial coverage of PEP.

Publications were excluded when PEP appeared only in the background, indexing terms, or an undifferentiated list of ERCP-related adverse events. Studies limited to other ERCP complications, treatment after PEP onset, non-ERCP pancreatitis, animal or cellular experiments, or unrelated topics were also excluded. Retracted publications and residual duplicates were removed.

Two reviewers (XD and CY) independently screened the records against the prespecified eligibility criteria. Disagreements were resolved through discussion, with consultation of a third reviewer (JT) when necessary.

### 2.4 Metadata and author-keyword normalization

Author names, institutions, countries or regions, source-journal titles, and author keywords were standardized using manually verified thesaurus files. Differences in capitalization, punctuation, spelling, abbreviations, and singular or plural forms were harmonized. Author-name variants were merged only when affiliation and publication information supported the same identity.

Keyword analyses were restricted to author-provided keywords to characterize topics explicitly identified by the authors and to use the same metadata category across WoSCC and Scopus. Keywords Plus and other database-generated index terms were excluded because they represent a different source of terminology and reflect database-specific indexing procedures. Combining these terms with author keywords would therefore mix different metadata categories rather than provide an equivalent replacement for missing author keywords. Publications without author keywords remained eligible for analyses not requiring keyword metadata. This restriction improves consistency of keyword provenance but does not resolve missing-data bias, particularly the lower availability of author keywords in earlier publications.

Keyword variants were standardized using a VOSviewer thesaurus file. Variants of “endoscopic retrograde cholangiopancreatography” were merged as “ERCP,” and PEP variants were standardized as “post-ERCP pancreatitis.” Synonymous terms were consolidated only when they represented the same concept, whereas conceptually distinct terms were retained. General or non-informative standalone keywords were removed according to explicit normalization rules documented in the Supplementary Material.

### 2.5 Bibliometric and knowledge-mapping analyses

The merged WoSCC–Scopus dataset (*n* = 1,686) was used to analyze annual output, source journals, contributing countries or regions, institutions, authors, collaboration networks, and author-keyword patterns. Citation-dependent analyses, including cited-journal, cited-author, and cited-reference co-citation networks and reference citation bursts, were based on the 1,449 included WoSCC records. The 237 Scopus-unique publications were retained in the merged dataset for descriptive and author-keyword analyses but did not contribute their reference lists to the co-citation networks. Restricting these networks to WoSCC provided a consistent source of cited-reference metadata and avoided additional cross-database reference harmonization. This choice does not eliminate database-coverage bias. Scopus-unique publications could still appear as cited-reference nodes if cited by the included WoSCC records and retained under the network selection criteria. Citation counts and cited-reference fields from the two databases were not pooled. The merged dataset covered 1975–2026, whereas the WoSCC records used for reference analyses covered 1985–2026.

Annual publication output and source-journal productivity were summarized using the bibliometrix package (version 5.0) in R version 4.6.0 (10). The same environment was used to rank and visualize the 10 most productive source journals. The exponential trend was fitted using complete-year data from 1975 to 2025; the partial 2026 data were excluded from model fitting.

VOSviewer version 1.6.20 was used to construct country and institutional collaboration networks, author co-authorship networks, author-keyword co-occurrence networks, and cited-journal and cited-author co-citation networks (11). Full counting and association-strength normalization were applied. Full counting was used to describe participation in publications and collaboration networks rather than to allocate proportional research credit among participating entities. Total link strength (TLS) quantified the aggregate collaboration or co-occurrence links of each item. Minimum inclusion thresholds are reported in the corresponding figure legends, whereas the numbers of eligible and displayed items are reported in the Results.

CiteSpace version 7.0.1 was used for reference co-citation analysis, keyword clustering, temporal visualization, and citation-burst detection (12). Analyses used one-year time slices and the g-index with k = 25. Pathfinder pruning was applied to the reference co-citation network to remove redundant links and facilitate interpretation of its structural backbone. Keyword clusters were labeled using the log-likelihood ratio algorithm and generated without additional network pruning to retain co-occurrence relationships among the selected keyword nodes, including connections between thematic groups. For a fixed node set, removing links through pruning lowers network density and may alter connectivity and cluster boundaries. The unpruned keyword network retains these relationships but may present a more interconnected structure. Network densities were therefore not directly compared across the two analyses. Keyword and reference bursts were summarized by burst strength and duration.

### 2.6 Reproducibility and data management

Screening decisions and exclusion reasons were recorded in a structured audit file. The analysis-ready dataset retained the title, DOI or other persistent identifier, publication year, document type, database source, and standardized author, institution, country, journal, and author-keyword fields. The complete search strategies, eligibility criteria, normalization rules, VOSviewer thesaurus file, and permitted analysis-ready data are provided in the Supplementary Material. Raw WoSCC and Scopus exports were not redistributed because of database licensing restrictions.

## 3 Results

### 3.1 Study selection

The searches identified 2,743 records in WoSCC and 2,104 in Scopus. After restriction to English-language articles and reviews, 1,886 WoSCC and 1,638 Scopus records remained, yielding 3,524 records for merging. CiteSpace removed 1,290 duplicates, followed by 68 additional duplicates identified manually.

Of the 2,166 records screened for topical relevance, 480 were excluded. These comprised 299 records in which PEP was absent from substantive title or abstract content or appeared only as an indexing or exclusion term; 151 records in which PEP was mentioned only as background or as an incidental or ancillary adverse event; 3 studies limited to treatment or management after PEP onset; 14 case reports or series outside the prespecified scope; 12 preclinical or mechanistic studies without an eligible clinical PEP focus; and 1 retracted publication. The final dataset comprised 1,686 publications, including 1,414 articles and 272 reviews. Of these, 1,449 were retained from WoSCC and 237 were unique to Scopus (Figure 1).

**Figure 1:**
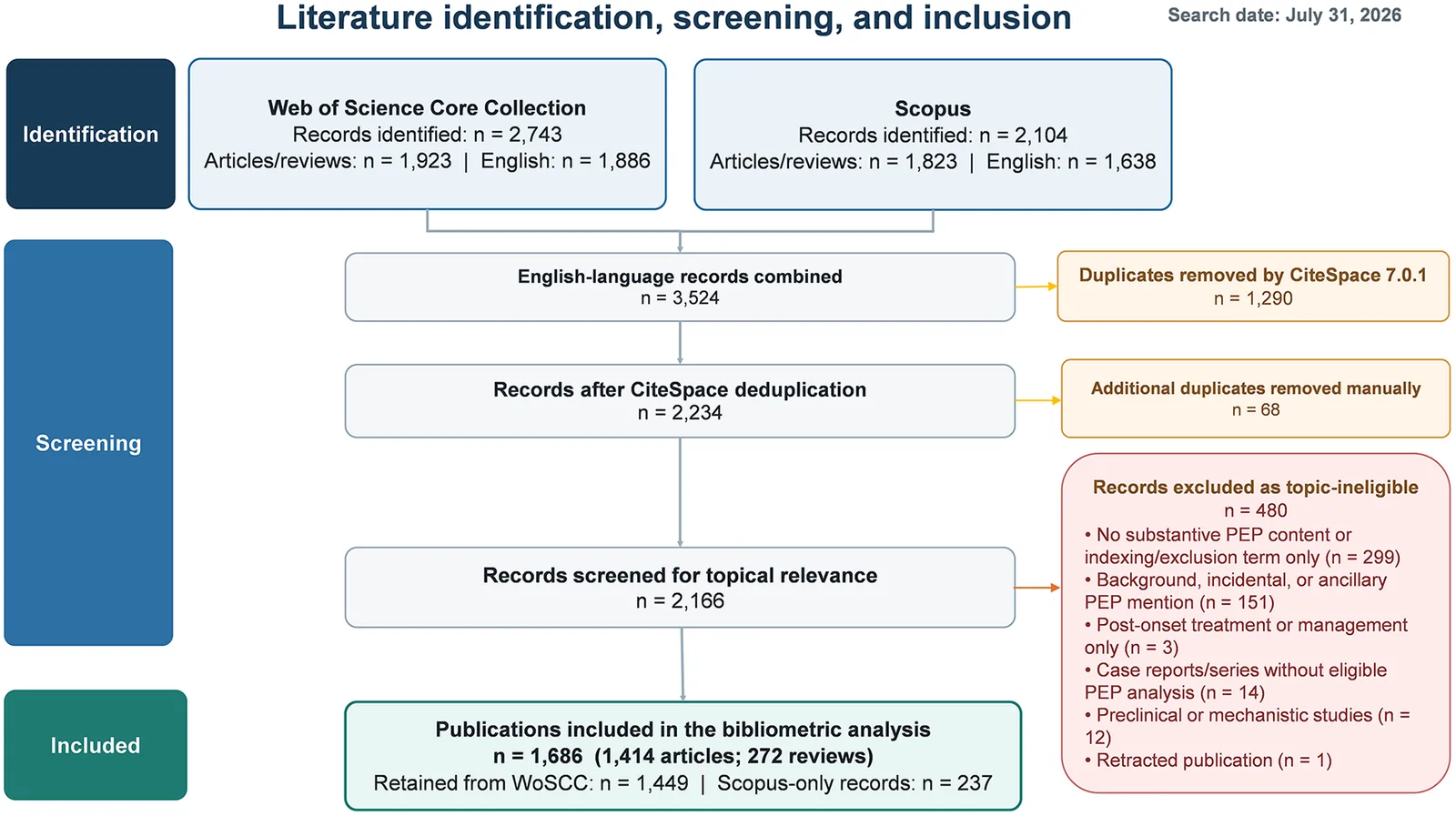
PRISMA-style flow diagram showing literature identification, deduplication, topical screening, and inclusion from WoSCC and Scopus. Mutually exclusive primary reasons for exclusion during topical screening are shown in the diagram.

### 3.2 Annual publication output

The 1,686 publications covered the period from 1975 to July 31, 2026. Annual output remained sporadic through 1995 and increased to 26 publications in 1996. Publication output reached 80 in 2015 and, after fluctuations during 2016–2019, increased from 89 in 2020 to a peak of 133 in 2025. A further 118 publications had been indexed by July 31, 2026. An exponential model fitted to complete-year data from 1975 to 2025 yielded an R^2^ of 0.943 (Figure 2).

**Figure 2:**
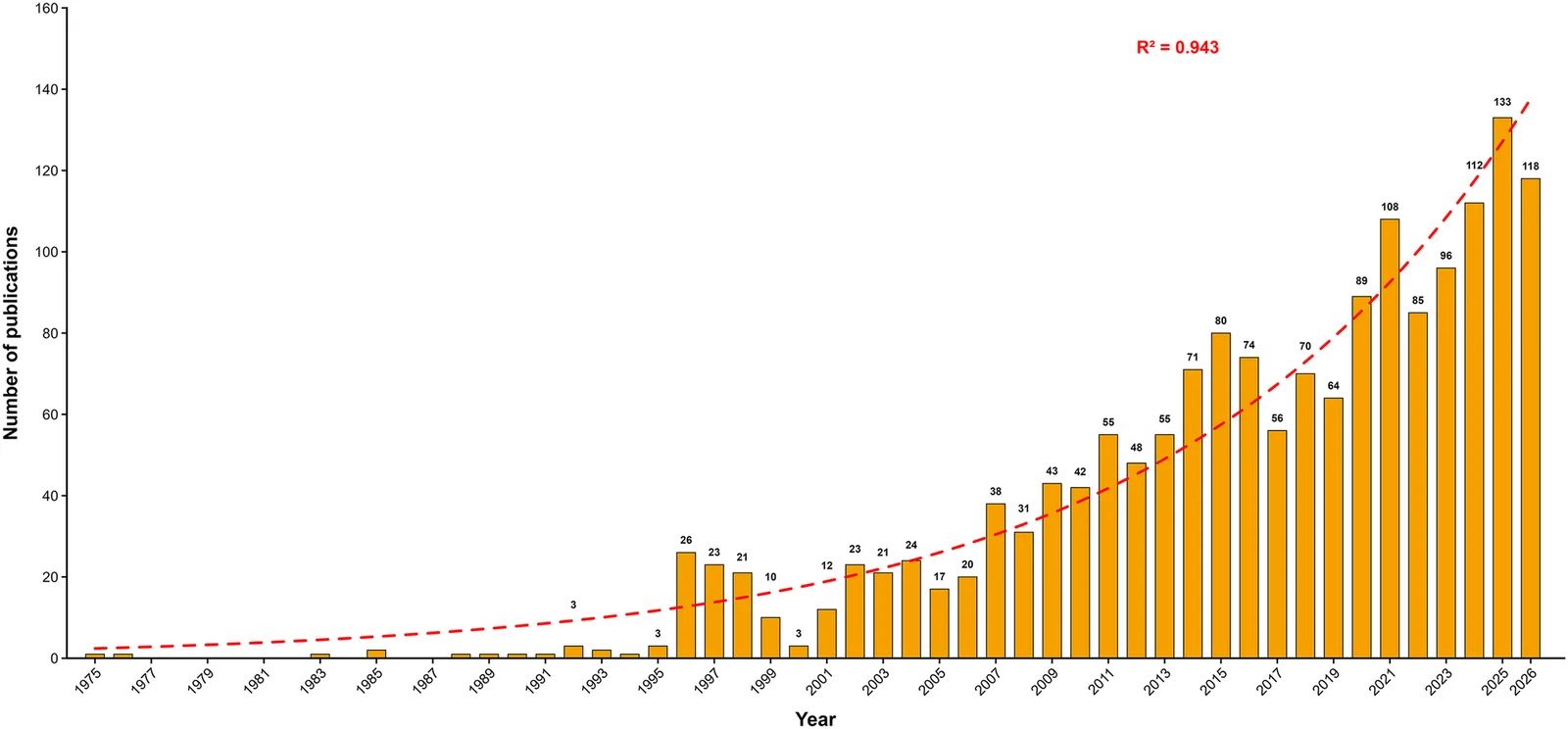
Annual publication output in post-ERCP pancreatitis research, 1975–2026. Data were derived from the merged WoSCC–Scopus dataset. The dashed red curve represents the exponential trend fitted to complete-year data from 1975 to 2025. Records indexed from January 1 to July 31, 2026, are shown but were excluded from model fitting.

### 3.3 Contributions and collaboration by country and institution

The included publications involved authors from 99 countries or regions. Of these, 40 met the minimum threshold of at least five publications, and 39 connected countries or regions were displayed in the collaboration network, which resolved into three clusters. The United States ranked first in publication output with 485 publications (28.8%), followed by China (306; 18.1%) and Japan (217; 12.9%). The United States maintained co-authorship links with countries in each of the three clusters (Figure 3A; Table 1).

**Figure 3:**
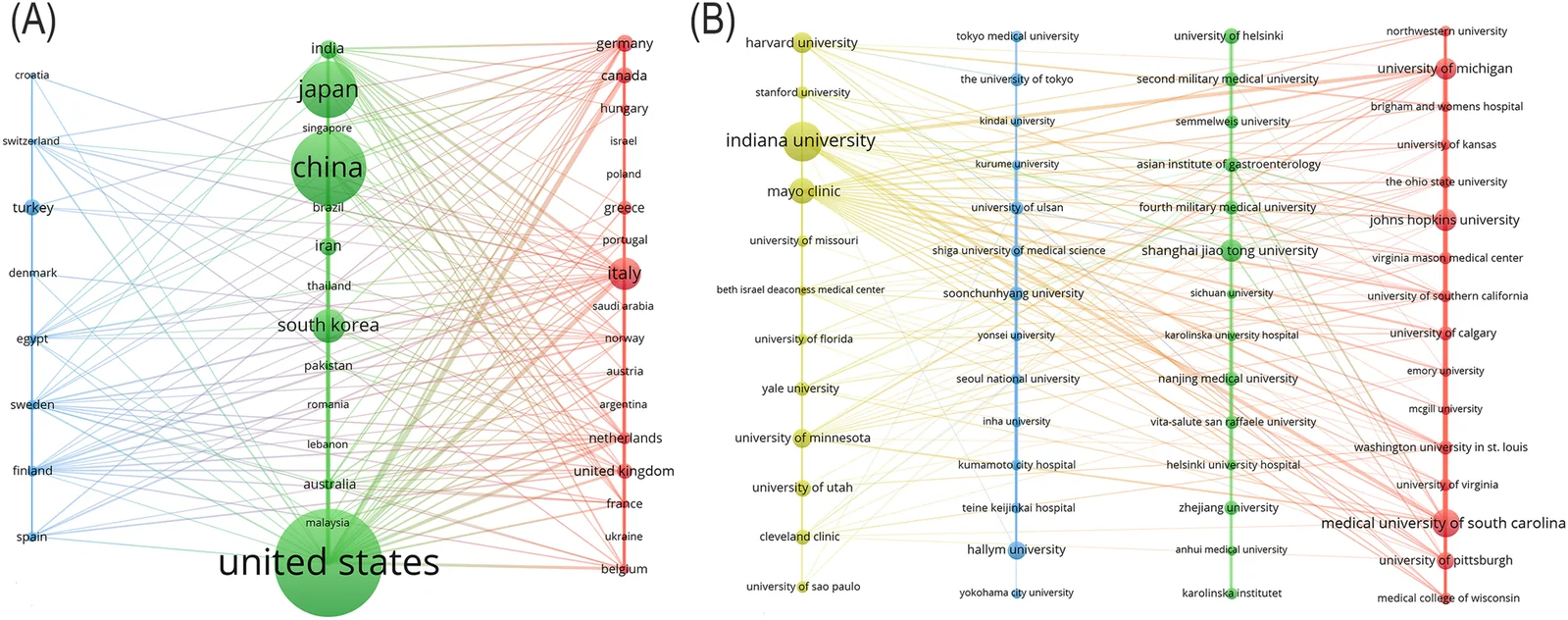
Country and institutional collaboration networks in post-ERCP pancreatitis research. Networks were constructed in VOSviewer from the merged WoSCC–Scopus dataset using full counting and association-strength normalization. **(A)** Country or region co-authorship network, with a minimum inclusion threshold of five publications. **(B)** Institutional co-authorship network, with a minimum inclusion threshold of 10 publications. Node size represents publication output, link thickness represents collaboration strength, and node color indicates cluster membership.

**Table 1:** Top 10 contributing countries and institutions in post-ERCP pancreatitis research.

Rank	Country	Documents *n* (%)	Rank	Institution	Documents *n* (%)
1	United States	485 (28.8%)	1	Indiana University	54 (3.2%)
2	China	306 (18.1%)	2	Medical University of South Carolina	36 (2.1%)
3	Japan	217 (12.9%)	3	Mayo Clinic	32 (1.9%)
4	South Korea	113 (6.7%)	4	Johns Hopkins University	27 (1.6%)
5	Italy	107 (6.3%)	5	Shanghai Jiao Tong University	27 (1.6%)
6	India	54 (3.2%)	6	University of Michigan	26 (1.5%)
7	Iran	51 (3.0%)	7	Harvard University	24 (1.4%)
8	Germany	48 (2.8%)	8	University of Minnesota	22 (1.3%)
9	Canada	45 (2.7%)	9	Hallym University	21 (1.2%)
10	Turkey	45 (2.7%)	10	University of Pittsburgh	20 (1.2%)

Percentages were calculated using the 1,686 included publications as the denominator. Under full counting, a publication involving authors from multiple countries or institutions was credited once to each participating entity; percentages therefore do not sum to 100%.

A total of 2,427 institutions contributed to the included literature. Fifty-nine institutions met the threshold of at least 10 publications, of which 56 connected institutions were displayed in the network and grouped into four clusters. Indiana University was the most productive institution with 54 publications (3.2%), followed by the Medical University of South Carolina (36; 2.1%) and Mayo Clinic (32; 1.9%) (Figure 3B; Table 1).

### 3.4 Source journals and journal co-citation

The 1,686 publications were distributed across 298 source journals. *Gastrointestinal Endoscopy* was the leading publication venue with 170 publications, followed by *Endoscopy* (*n* = 74) and *Surgical Endoscopy* (*n* = 67) (Figure 4A).

**Figure 4:**
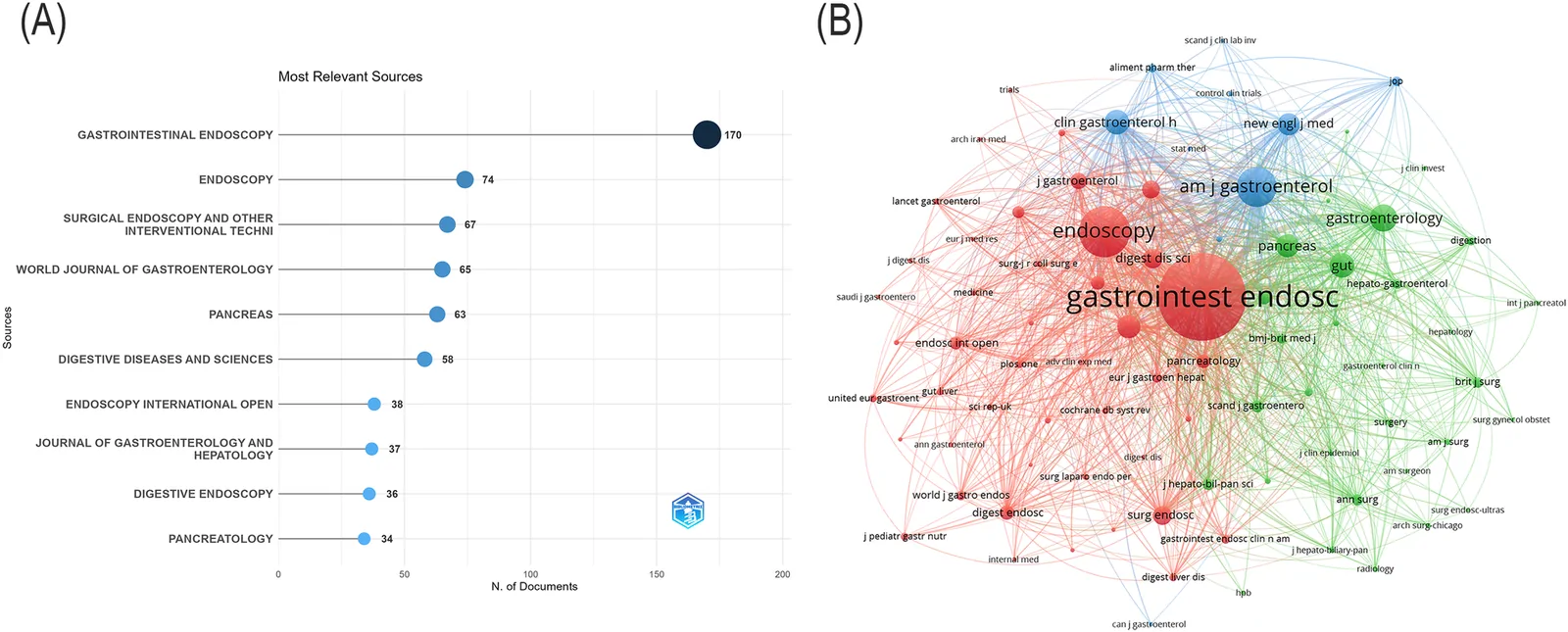
Source-journal productivity and cited-journal co-citation patterns in post-ERCP pancreatitis research. **(A)** Top 10 source journals ranked by publication output in the merged WoSCC–Scopus dataset and visualized using the bibliometrix package in R; bar length represents publication output. **(B)** Cited-journal co-citation network constructed in VOSviewer from the 1,449 WoSCC records, with a minimum inclusion threshold of 50 citations. Node size represents citation frequency, link thickness represents co-citation strength, and node color indicates cluster membership.

The reference lists of the 1,449 WoSCC records contained 2,579 cited journals. Of these, 84 received at least 50 citations, and all 84 were included in the connected co-citation network, which resolved into three clusters. *Gastrointestinal Endoscopy* was cited 11,065 times and had the highest total co-citation link strength (TLS=310,271), followed by *Endoscopy* with 4,468 citations (Figure 4B).

### 3.5 Author productivity, collaboration, and co-citation

The merged dataset included 8,175 unique authors. Of these, 40 met the minimum threshold of at least 10 publications, and 23 connected authors were displayed in the co-authorship network, which resolved into three clusters. Sherman S was the most productive author with 47 publications and the highest total co-authorship link strength (TLS = 122), followed by Lehman GA with 42 publications (TLS = 117) and Fogel EL with 32 publications (TLS = 100) (Figure 5A; Table 2).

**Figure 5:**
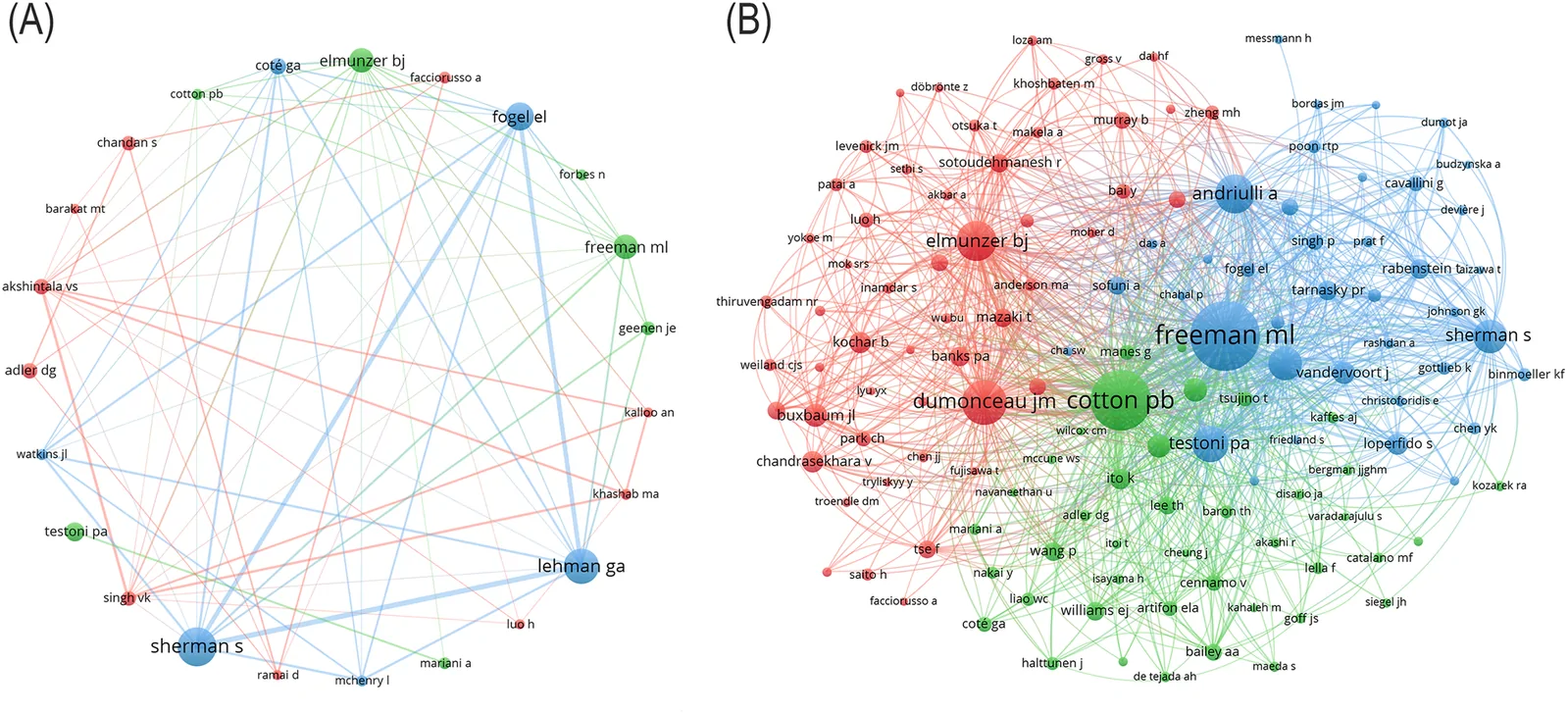
Author co-authorship and cited-author co-citation networks in post-ERCP pancreatitis research. Networks were constructed in VOSviewer using full counting and association-strength normalization. **(A)** Author co-authorship network based on the merged WoSCC–Scopus dataset, with a minimum inclusion threshold of 10 publications. Node size represents publication output, and link thickness represents co-authorship strength. **(B)** Cited-author co-citation network based on the 1,449 WoSCC records, with a minimum inclusion threshold of 50 citations. Node size represents citation frequency, and link thickness represents co-citation strength. In both panels, node color indicates cluster membership.

**Table 2:** Top 10 most productive authors and their total link strengths in post-ERCP pancreatitis research.

Rank	Author	Documents	Total link strength
1	Sherman S	47	122
2	Lehman GA	42	117
3	Fogel EL	32	100
4	Elmunzer BJ	28	50
5	Freeman ML	28	40
6	Testoni PA	21	8
7	Coté GA	18	52
8	Adler DG	17	12
9	Akshintala VS	16	45
10	Chandan S	15	18

TLS was calculated within the thresholded author co-authorship network; authors without links to other eligible authors were not displayed.

The reference lists of the 1,449 WoSCC records contained 9,279 cited authors. Of these, 135 received at least 50 citations, and 132 connected cited authors were displayed in the co-citation network, which resolved into three clusters. Freeman ML was the most frequently cited author, with 1,671 citations and a total co-citation link strength of 29,401, followed by Cotton PB and Dumonceau JM with 1,309 and 782 citations, respectively (Figure 5B).

### 3.6 Keyword co-occurrence, clustering, and temporal trends

Author keywords were available for 1,139 of the 1,686 included publications (67.6%). After normalization and removal of non-informative terms, 1,680 unique author keywords remained. Of these, 105 occurred at least five times, and all 105 were included in the connected VOSviewer co-occurrence network, which resolved into three broad clusters (Figure 6A). The most frequent terms were ERCP (*n* = 784), post-ERCP pancreatitis (*n* = 467), and pancreatitis (*n* = 342). Beyond these search-defining terms, the main thematic areas included cannulation techniques, pharmacological prophylaxis, pancreatic stenting, hydration, and risk assessment.

**Figure 6:**
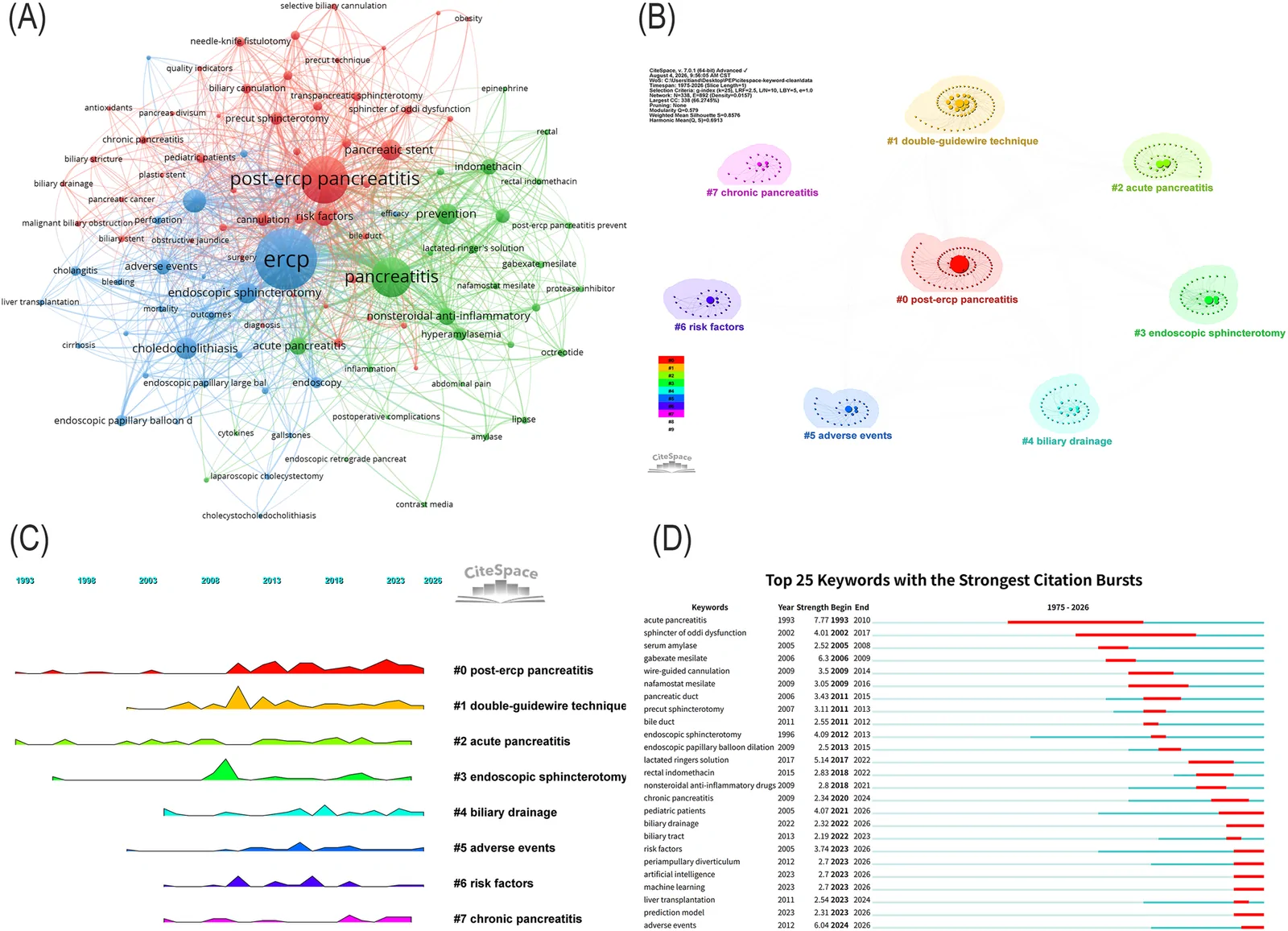
Author-keyword co-occurrence, clustering, and temporal trends in post-ERCP pancreatitis research. Analyses used cleaned author keywords from the merged WoSCC–Scopus dataset. **(A)** VOSviewer network of 105 keywords occurring at least five times, grouped into three clusters. Node size represents occurrence frequency, link thickness represents co-occurrence strength, and color indicates cluster membership. **(B)** CiteSpace time-sliced map comprising eight clusters (1-year slices; g-index, k = 25; log-likelihood ratio labeling; no pruning; modularity Q = 0.579; silhouette S = 0.858). Cluster counts in panels A and B are not directly comparable because different clustering procedures were used. **(C)** Keyword-cluster activity during 1993–2026; 1993 was the first year with usable author-keyword data. **(D)** Top 25 keyword bursts; red segments indicate burst periods. Data for 2026 extend through July 31.

The CiteSpace time-sliced analysis generated eight more granular keyword clusters, of which #0 post-ERCP pancreatitis and #1 double-guidewire technique were the largest (Figure 6B). Because VOSviewer and CiteSpace used different node-selection and clustering procedures, the numbers of clusters generated by the two analyses are not directly comparable. The CiteSpace clustering solution had a modularity Q of 0.579 and a weighted mean silhouette of 0.858, suggesting reasonable separation and internal consistency among the generated clusters.

The temporal map showed earlier prominence of disease- and technique-related topics, followed by the later emergence of hydration and risk-oriented themes. The double-guidewire technique and endoscopic sphincterotomy clusters showed their main activity around 2010, whereas lactated Ringer's solution showed a later occurrence burst from 2017 to 2022 (strength = 5.14) (Figure 6C).

Recent bursts were concentrated in risk assessment and procedural safety. Adverse events showed the strongest ongoing burst (strength = 6.04; 2024–2026), followed by risk factors (strength = 3.74; 2023–2026). Artificial intelligence and machine learning also showed emerging bursts from 2023 to 2026, although their burst strengths were lower (2.70 each) (Figure 6D).

### 3.7 Reference co-citation and citation bursts

The reference co-citation network constructed from the 1,449 WoSCC records comprised 1,127 nodes and 5,693 links, with a network density of 0.009. The largest connected component accounted for 82.9% of all nodes (Figure 7A).

**Figure 7:**
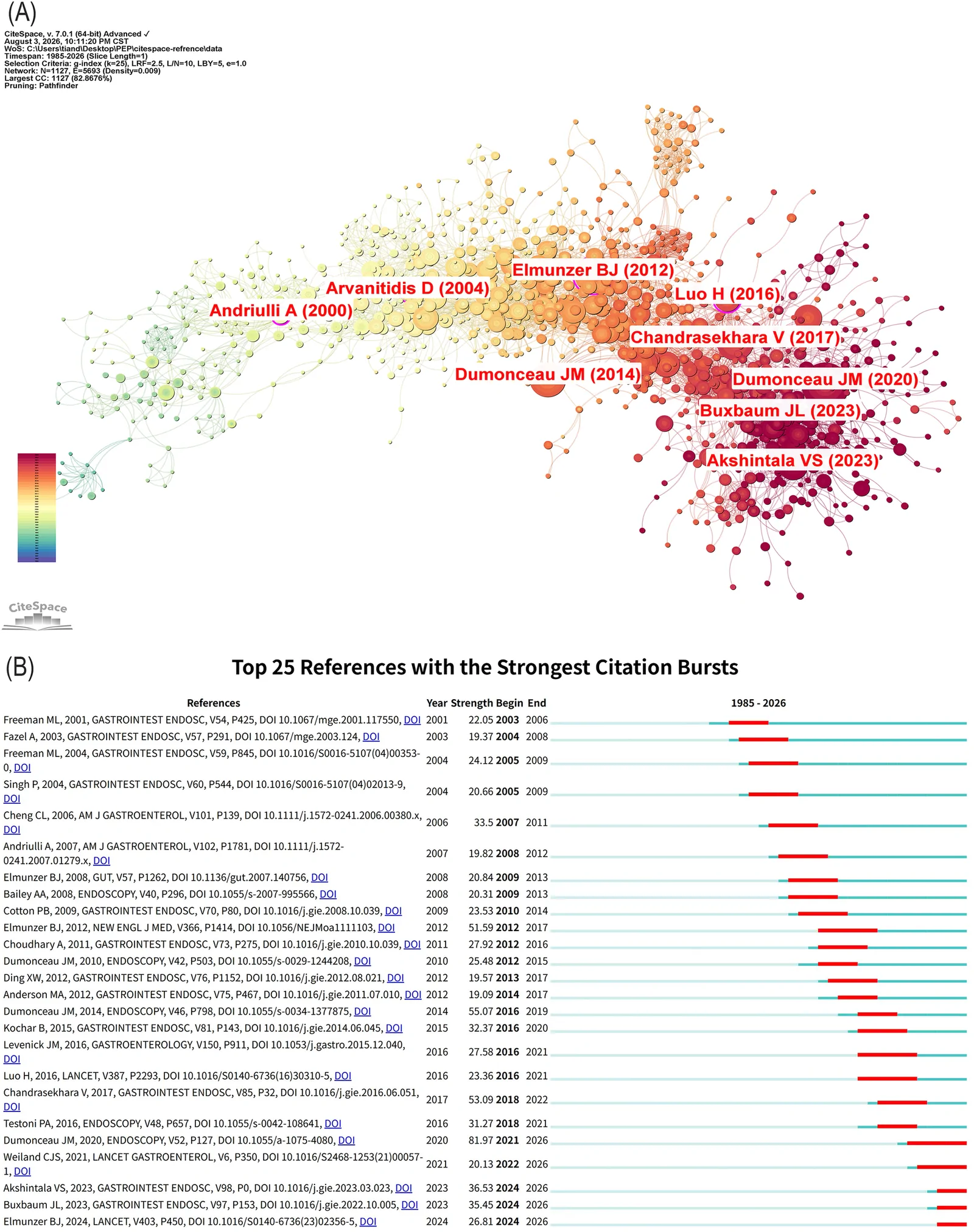
Reference co-citation structure and citation bursts in post-ERCP pancreatitis research. Analyses were based on the 1,449 WoSCC records covering 1985–2026. **(A)** CiteSpace reference co-citation network generated using 1-year time slices, the g-index (k = 25), and Pathfinder pruning. Node size represents co-citation frequency, links represent co-citation relationships, and node and link colors indicate the time slices in which citations occurred. Labels identify selected references by first author and publication year. **(B)** Top 25 references with the strongest citation bursts; red segments indicate burst periods. Data for 2026 cover January 1 to July 31.

Among the most frequently co-cited references, Cotton et al. (1991) (13) ranked first with 733 co-citations, followed by Freeman et al. (2001; *n* = 453) and Freeman et al. (1996; *n* = 452) (Table 3).

**Table 3:** Top 10 most frequently co-cited references in post-ERCP pancreatitis research.

Rank	Article title	First author	Year	Journal	Co-citations
1	Endoscopic sphincterotomy complications and their management: an attempt at consensus (13)	Cotton PB	1991	Gastrointest Endosc	733
2	Risk factors for post-ERCP pancreatitis: a prospective, multicenter study (3)	Freeman ML	2001	Gastrointest Endosc	453
3	Complications of endoscopic biliary sphincterotomy (14)	Freeman ML	1996	N Engl J Med	452
4	A randomized trial of rectal indomethacin to prevent post-ERCP pancreatitis (15)	Elmunzer BJ	2012	N Engl J Med	310
5	Prophylaxis of post-ERCP pancreatitis: European Society of Gastrointestinal Endoscopy (ESGE) Guideline—updated June 2014 (16)	Dumonceau JM	2014	Endoscopy	261
6	Incidence rates of post-ERCP complications: a systematic survey of prospective studies (17)	Andriulli A	2007	Am J Gastroenterol	256
7	Risk factors for post-ERCP pancreatitis: a prospective multicenter study (18)	Cheng CL	2006	Am J Gastroenterol	253
8	ERCP-related adverse events: European Society of Gastrointestinal Endoscopy (ESGE) Guideline (19)	Dumonceau JM	2020	Endoscopy	244
9	Incidence, severity, and mortality of post-ERCP pancreatitis: a systematic review by using randomized, controlled trials (20)	Kochar B	2015	Gastrointest Endosc	234
10	Complications of diagnostic and therapeutic ERCP: a prospective multicenter study (21)	Masci E	2001	Am J Gastroenterol	231

References are ranked by co-citation frequency in the WoSCC-based reference co-citation analysis.

Prominent citation bursts during 2012–2022 were associated with preventive evidence and practice guidance. Dumonceau et al. (2014) (16) showed the strongest burst within this period (strength = 55.07; 2016–2019), followed by Chandrasekhara et al. (2017; strength = 53.09; 2018–2022) and Elmunzer et al. (2012; strength = 51.59; 2012–2017) (Figure 7B).

Recent burst activity remained centered on updated prevention guidance and evidence synthesis. Dumonceau et al. (2020) (19) showed the strongest overall burst (strength = 81.97; 2021–2026). Akshintala et al. (2023) (2) and Buxbaum et al. (2023) (4) showed ongoing bursts during 2024–2026, with strengths of 36.53 and 35.45, respectively.

## 4 Discussion

Across the included literature, PEP research developed from a field organized around complication definition and procedural risk into one increasingly concerned with prevention and risk prediction. The mapping also showed that this development was not geographically or intellectually diffuse: collaboration remained centered on established endoscopy networks, while consensus papers, multicenter risk studies, prevention trials, and guidelines formed much of the co-citation structure. These patterns describe how the literature was organized and used; they do not rank interventions or establish comparative efficacy.

### 4.1 Evolution of PEP research

Temporal mapping showed an early focus on disease recognition and procedure-related risk, followed by later attention to hydration, multimodal prevention, and risk-stratified care. The continuing clinical relevance of this work is supported by an updated meta-analysis of 145 randomized trials, which estimated PEP incidences of 10.2% in unselected patients and 14.1% in high-risk patients and found no significant decline in control-group incidence across the study period (2). Earlier prospective evidence likewise identified pancreatitis as the most frequent major complication after ERCP (17). Publication growth should therefore be read as increasing research activity, not as evidence that the population incidence of PEP has risen.

Several developments may have contributed to the growth in publications. These include the standardization of PEP definitions and severity grading, influential randomized trials and successive society guidelines on prophylaxis, the shift from isolated preventive measures toward multimodal strategies, and more recent interest in multivariable and artificial intelligence–assisted risk prediction (13, 15, 16, 19, 22–27). Increasing contributions from East Asia and the development of multicenter research networks may also have expanded research capacity in this field. These explanations are consistent with the temporal, geographic, and co-citation patterns observed in the present study, but they should be regarded as plausible interpretations because bibliometric data cannot establish the causes of publication growth.

### 4.2 Geographic and collaborative structure

The collaboration maps remained centered on endoscopy networks in the United States, alongside substantial participation from East Asia. This pattern has a historical counterpart in influential multicenter studies that defined patient- and procedure-related risk (3, 14) and in prevention trials conducted through North American referral centers (15). Network position, however, reflects research participation and collaboration rather than clinical representativeness. Differences in case mix, operator experience, indications, and prophylactic practice between high-volume referral centers and routine-care settings should therefore be considered when interpreting the geographical reach of the evidence.

### 4.3 Disciplinary concentration and the foundational knowledge base

The journal and reference co-citation maps point to a clinical knowledge base strongly anchored in specialist endoscopy literature. Cotton et al. provided an early consensus framework for defining and grading ERCP-related complications (13), while multicenter studies by Freeman and colleagues linked PEP risk to both patient characteristics and procedural factors (3, 14). This terminology framework was later expanded and standardized through the American Society for Gastrointestinal Endoscopy (ASGE) lexicon for endoscopic adverse events (28). More recently, an international cohort study found that the revised Atlanta classification more accurately reflected PEP severity than the traditional consensus grading system, which relies heavily on the duration of hospitalization (29). The persistent co-citation of the early consensus and risk-factor studies is understandable because subsequent research required consistent outcome definitions and a shared vocabulary for describing risk.

### 4.4 Development of evidence-based prevention

The keyword structure brought pharmacological prophylaxis, pancreatic stenting, and cannulation-related measures into the same connected literature. Their proximity in the keyword network represents a shared research focus and does not establish the superiority of any particular combination. The strong citation burst of Elmunzer et al. (strength = 51.59; 2012–2017) coincided with the period in which rectal nonsteroidal anti-inflammatory drugs (NSAIDs) became established in PEP prevention. Their randomized trial demonstrated benefit in high-risk patients (15), followed by the incorporation of rectal NSAIDs into the European Society of Gastrointestinal Endoscopy (ESGE) guidelines (16).

Procedural prevention evolved in parallel. Prophylactic pancreatic stenting was supported by meta-analytic evidence in high-risk patients (30), while guidance on biliary access emphasized wire-guided cannulation and limits on repeated papillary manipulation (31). The prominence of the double-guidewire technique in the keyword clusters reflects sustained interest in rescue biliary access after unintended pancreatic duct cannulation (32). Comparative evidence, however, has not consistently shown this technique to be superior to other rescue approaches, so its bibliometric prominence should not be interpreted as evidence of clinical preference. More recent evidence has refined rather than displaced the role of stenting. In a 2024 randomized non-inferiority trial, PEP occurred in 14.9% of patients receiving indomethacin alone and 11.3% of those receiving indomethacin plus a prophylactic pancreatic stent; indomethacin alone did not meet the criterion for non-inferiority (23). The result is consistent with a risk-adapted approach in which pharmacological and procedural measures are not assumed to be interchangeable.

Hydration entered the prevention literature later, matching its later position in the temporal map. A multicenter randomized trial found a lower PEP rate with aggressive lactated Ringer's hydration than with standard hydration (33). However, the FLUYT trial did not demonstrate additional benefit when aggressive hydration was added to routine rectal NSAID prophylaxis (34). A subsequent network meta-analysis brought NSAIDs, intravenous fluids, pancreatic stents, and their combinations into a single comparative framework, although confidence in most comparisons was low to moderate (35). ESGE and ASGE guidance subsequently incorporated hydration alongside NSAIDs, cannulation strategy, and pancreatic stenting, although its place in the prevention pathway differs according to concomitant prophylaxis, patient risk, and the potential for fluid overload (4, 19). These findings are consistent with the increasing bibliometric prominence of multimodal prevention, but they do not establish that every combination provides additional benefit in all patients.

### 4.5 Risk stratification and emerging analytical tools

The recent appearance of risk-oriented terms alongside artificial intelligence and machine learning marks a move from population-level risk factors toward patient-level prediction. A recent meta-analysis based on adjusted estimates identified multiple patient- and procedure-related predictors of PEP, providing a rationale for multivariable rather than single-factor prediction approaches (24). A prospective multicenter model based on routinely available clinical and procedural variables achieved internally validated discrimination of approximately 0.79 but did not undergo independent external validation (25). An imaging-based model using duodenal papilla radiomics reported higher discrimination in its development and validation cohorts (26). Both approaches showed potential, but their generalizability across clinical settings remains uncertain.

A recent systematic review of PEP prediction models reached a similar conclusion: external validation remained limited, and the risk of bias was substantial in many studies (27). The emerging keyword bursts should therefore be interpreted as evidence of increasing research activity rather than clinical maturity. Before clinical implementation, prediction models require independent external validation, assessment of calibration and clinical utility, and transparent handling of missing data. TRIPOD + AI provides an appropriate reporting framework for both regression- and machine-learning-based prediction studies (22).

### 4.6 Implications for research and clinical translation

Beyond PEP, these findings illustrate how research on a procedure-related adverse event can progress from complication definition and risk identification toward combined prevention and individualized prediction. For investigators and research funders, bibliometric mapping can show where evidence production is concentrated and where geographic or institutional participation remains limited. The convergence of pharmacological, device-based, procedural, and prediction-related themes points to the need to evaluate integrated prevention pathways rather than isolated measures. Such maps can inform research priorities and collaboration, but they cannot determine treatment efficacy or replace evidence from clinical trials, systematic reviews, and practice guidelines.

The concentration of publication output in North America and East Asia limits the extent to which the observed research priorities can be assumed to represent those of low- and middle-income countries (LMICs). Geographic location alone, however, does not establish a country's income level or an institution's resources. The prominence of pancreatic stenting or intensive hydration in the literature does not demonstrate their feasibility across health systems, where implementation may depend on endoscopic expertise, equipment availability, staffing, and monitoring capacity. These factors were not assessed in this study. The maps should therefore inform questions for locally relevant research rather than prescribe uniform prevention priorities. Studies involving resource-constrained settings are needed to evaluate feasibility, affordability, and implementation alongside clinical outcomes.

### 4.7 Strengths and limitations

The dual-database design broadened coverage of publication, collaboration, and author-keyword patterns, while cross-database deduplication and manual normalization reduced avoidable fragmentation. Restricting keyword analyses to author-provided terms limited contamination by database-generated index terms. Citation-dependent analyses were confined to WoSCC records, avoiding direct pooling of citation fields produced by different databases. However, the reference lists of the 237 Scopus-unique publications (14.1% of the merged dataset) did not contribute to these networks. Including them could change co-citation frequencies, link strengths, and the representation of references and thematic clusters. Their possible presence as cited-reference nodes would not recover the relationships contributed by their own reference lists. The magnitude of this effect was not quantified, and the co-citation findings should therefore be interpreted as a WoSCC-based representation of the field rather than a complete dual-database network.

Several limitations remain. WoSCC and Scopus differ in journal coverage, cited-reference completeness, and metadata structure, and conversion to a common format cannot remove all source-specific heterogeneity. English-language restriction may have reduced the visibility of regional literature. Author and institutional disambiguation may remain incomplete. Incomplete author-keyword coverage limits the temporal interpretation of Figure 6, particularly for publications before 1993. Topics discussed in records without author keywords cannot contribute to the keyword maps, potentially underrepresenting early research and making some themes appear more recent than their underlying literature. Temporal patterns may therefore reflect both changes in research attention and changes in metadata availability. Restricting the analysis to author keywords may favor the representation of recent publications, but the magnitude of this bias was not quantified. Sparse early keyword activity should not be interpreted as an absence of research, and keyword appearance or burst dates do not establish when a topic first emerged.

Full counting assigns one publication credit to each participating country or institution, irrespective of its role. Compared with fractional allocation, this may increase the apparent contribution of entities participating in multinational publications, including secondary partners. Our data did not distinguish leading from secondary partners or quantify their substantive contributions. The reported counts therefore indicate participation rather than leadership or proportional contribution. Fractional counting offers an alternative weighting approach but does not itself establish actual contributions. The sensitivity of country and institutional rankings to the counting method was not assessed.

The search strategy and eligibility criteria focused on clinically oriented research on PEP incidence, risk assessment, prediction, and prevention. Requiring the second concept block may have reduced retrieval of publications framed primarily around mechanisms or post-onset management. In addition, animal or cellular experiments and studies limited to treatment after PEP onset were outside the prespecified eligibility criteria. The findings should therefore be interpreted within this clinical scope rather than as a comprehensive map of all PEP research. Node-selection thresholds and pruning settings can influence network connectivity and the visibility of smaller topics. The sensitivity of the network structures to alternative pruning settings was not assessed. Co-citation also favors work with longer citation exposure, and citation bursts measure attention rather than validity. Data for 2026 were incomplete at the July 31 cutoff. Finally, this study mapped research structure and development; it did not estimate intervention effects or assess the risk of bias of the underlying clinical evidence.

## 5 Conclusion

This dual-database analysis shows that PEP research has shifted from complication definition and procedural risk toward multimodal prevention and individualized prediction. Research output has expanded markedly, with strong contributions from East Asia, although collaboration and citation structures remain concentrated in established endoscopy networks and specialist journals. The resulting maps highlight gaps in geographic and institutional participation and areas requiring further clinical evaluation, but bibliometric prominence should not be interpreted as evidence of study quality or treatment efficacy.

## Data Availability

The original contributions presented in the study are included in the article/Supplementary Material, further inquiries can be directed to the corresponding author.
