# Supplementary material for: Research trends in post-ERCP pancreatitis: a dual-database bibliometric analysis
Source: Front Med (Lausanne). 2026 Oct 2;13:1966046. doi: 10.3389/fmed.2026.1966046 (PMC13671983; doi:10.3389/fmed.2026.1966046)
Supplement: Supplementary file 1 [file Table1.docx]

**Supplementary Material**

**Supplementary Tables**

**Supplementary Table S1.** Search strategies used in WoSCC and Scopus

| **Database** | **Search strategy** |
| --- | --- |
| WoSCC | TS= ("post-ERCP pancreatitis" OR "post ERCP pancreatitis" OR "post-endoscopic retrograde cholangiopancreatography pancreatitis" OR "postendoscopic retrograde cholangiopancreatography pancreatitis" OR "ERCP-induced pancreatitis" OR "ERCP related pancreatitis" OR "ERCP-related pancreatitis" OR "pancreatitis after ERCP" OR "pancreatitis following ERCP") AND TS= (risk* OR predict* OR stratifi* OR susceptib* OR incidence OR prevent* OR prophyl* OR "risk assessment" OR "risk score*" OR "risk model*" OR "prediction model*" OR "predictive model*" OR "machine learning" OR "artificial intelligence" OR "nonsteroidal anti-inflammatory drug*" OR "non-steroidal anti-inflammatory drug*" OR NSAID* OR indomethacin OR diclofenac OR naproxen OR "pancreatic duct stent*" OR "pancreatic stent*" OR hydration OR "aggressive hydration" OR "lactated Ringer*" OR "Ringer lactate" OR "Ringer's lactate" OR somatostatin OR octreotide OR gabexate OR nafamostat OR nitroglycerin OR "glyceryl trinitrate" OR cannulat* OR "guidewire technique*" OR "double-guidewire" OR "double guidewire" OR sphincterotom*) |
| Scopus | TITLE-ABS-KEY ( "post-ERCP pancreatitis" OR "post ERCP pancreatitis" OR "post-endoscopic retrograde cholangiopancreatography pancreatitis" OR "postendoscopic retrograde cholangiopancreatography pancreatitis" OR "ERCP-induced pancreatitis" OR "ERCP related pancreatitis" OR "ERCP-related pancreatitis" OR "pancreatitis after ERCP" OR "pancreatitis following ERCP" ) AND TITLE-ABS-KEY ( risk* OR predict* OR stratifi* OR susceptib* OR incidence OR prevent* OR prophyl* OR "risk assessment" OR "risk score*" OR "risk model*" OR "prediction model*" OR "predictive model*" OR "machine learning" OR "artificial intelligence" OR "nonsteroidal anti-inflammatory drug*" OR "non-steroidal anti-inflammatory drug*" OR NSAID* OR indomethacin OR diclofenac OR naproxen OR "pancreatic duct stent*" OR "pancreatic stent*" OR hydration OR "aggressive hydration" OR "lactated Ringer*" OR "Ringer lactate" OR "Ringer's lactate" OR somatostatin OR octreotide OR gabexate OR nafamostat OR nitroglycerin OR "glyceryl trinitrate" OR cannulat* OR "guidewire technique*" OR "double-guidewire" OR "double guidewire" OR sphincterotom* ) |
